# *Plasmodium vivax* Reticulocyte Binding Proteins for invasion into reticulocytes

**DOI:** 10.1111/cmi.13110

**Published:** 2019-09-08

**Authors:** Li‐Jin Chan, Melanie H. Dietrich, Wang Nguitragool, Wai‐Hong Tham

**Affiliations:** ^1^ The Walter and Eliza Hall Institute of Medical Research Parkville Victoria Australia; ^2^ Department of Medical Biology The University of Melbourne Melbourne Victoria Australia; ^3^ Department of Molecular Tropical Medicine and Genetics, Faculty of Tropical Medicine Mahidol University Bangkok Thailand

**Keywords:** antibodies, host–pathogen interactions, *Plasmodium falciparum*, *Plasmodium vivax*, structure biology

## Abstract

*Plasmodium vivax* is responsible for most of the malaria infections outside Africa and is currently the predominant malaria parasite in countries under elimination programs. *P. vivax* preferentially enters young red cells called reticulocytes. Advances in understanding the molecular and cellular mechanisms of entry are hampered by the inability to grow large numbers of *P. vivax* parasites in a long‐term in vitro culture. Recent progress in understanding the biology of the *P. vivax* Reticulocyte Binding Protein (PvRBPs) family of invasion ligands has led to the identification of a new invasion pathway into reticulocytes, an understanding of their structural architecture and PvRBPs as targets of the protective immune response to *P. vivax* infection. This review summarises current knowledge on the role of reticulocytes in *P. vivax* infection, the function of the PvRBP family of proteins in generating an immune response in human populations, and the characterization of anti‐PvRBP antibodies in blocking parasite invasion.

## 1 *Plasmodium vivax* INVASION OF RETICULOCYTES

Malaria parasites are exquisitely adapted for invasion into red blood cells. The merozoite, an ovoid‐shaped cell with an apical prominence, is the form of the malaria parasite that invades blood cells. Merozoites express parasite adhesins at the apical tip, where secretory organelles such as rhoptires and micronemes are present, which bind to specific red blood cell receptors to initiate a series of molecular events that commit the parasite to invasion and successful entry (Cowman, Tonkin, Tham, & Duraisingh, 2017; Tham, Beeson, & Rayner, 2017). After entry, the merozoite grows and replicates within the blood cell to produce 16–32 new merozoites that rupture out of the infected cell to invade other healthy red blood cells. This blood stage cycle of infection results in the clinical symptoms observed in malaria infection.

Understanding how malaria parasites recognize and enter blood cells provide opportunities to block invasion and stop the cycle of blood stage infection. There are six *Plasmodium* species that commonly infect humans: *Plasmodium falciparum*, *Plasmodium knowlesi*, *Plasmodium vivax*, *Plasmodium ovale curtisi*, *Plasmodium ovale wallikeri*, and *Plasmodium malariae*. *P*. *falciparum* and *P. vivax* are responsible for the majority of malaria infections in humans*. P. falciparum*, *P. knowlesi*, and *P. malariae* invade mature red blood cells called normocytes, although *P. falciparum* and *P. knowlesi* may also preferentially enter reticulocytes (Gruner et al., 2004; Lim et al., 2013; Moon et al., 2016). In contrast, *P. vivax* and *P. ovale* are more restricted in their host cell preference than *P. falciparum* and will generally invade reticulocytes. Since the establishment of a continuous in vitro culture for *P. falciparum* in the late 1970s, the field of malaria parasite invasion has been dominated by studies of *P. falciparum* invading normocytes. Collectively, these studies have provided insights into the step‐wise nature of parasite entry, have identified parasite and host factors involved in invasion, and led to the development of inhibitors and antibodies that can block parasite invasion and provide protection from clinical disease (Paul *et al*., 2015; Cowman et al., 2017; Draper *et al*., 2018). In contrast, *P. vivax* invasion into reticulocytes is poorly understood due to the lack of a long‐term in vitro culture system for this parasite species (Kanjee, Rangel, Clark, & Duraisingh, 2018; Tham et al., 2017).

The preference of *P. vivax* for reticulocytes has implications in infection dynamics, parasite reservoirs, and potential parasite killing mechanisms. There are two distinct classes of reticulocytes that are present within the bone marrow compartment and in peripheral circulation (Griffiths et al., 2012). In the bone marrow compartment, R1 reticulocytes that have expelled the nucleus, but retain residual reticulum and are motile and multi‐lobular. R2 reticulocytes are released from the bone marrow to the peripheral circulation and are non‐motile and mechanically stable. As these reticulocytes mature in the bone marrow and in peripheral circulation, they remove all their organelles and lose 20% of their plasma membrane surface area (Moras, Lefevre, & Ostuni, 2017). Reticulocytes express several surface proteins that are lost as they mature into normocytes. In particular, CD71 (Transferrin Receptor 1, TfR1), CD49d, CD151, CD81, and CD82 are present only on young reticulocytes compared with mature red blood cells (Thomson‐Luque et al., 2018). Using short‐term ex vivo cultures, *P. vivax* has been observed to have higher invasion rates into reticulocytes with high levels of TfR1 compared with reticulocytes with lower levels of TfR1 (Malleret et al., 2014). In the same study, *P. vivax* invasion into TfR1 high‐reticulocytes caused a more rapid loss of TfR1 and expulsion of the residual reticulum compared with uninfected reticulocytes. However, a study using Indian *P. vivax* strains showed large differences in reticulocyte preferences (Lim et al., 2016). Although there was a low prevalence of circulating schizonts (the mature replicative form of the parasite), there was an association between increased reticulocyte preference and the number of schizonts, suggesting a potential link between invasion of younger reticulocytes and effective parasite development. This study also showed the detection of early‐stage *P. vivax* infection in reticulocytes with visible reticulum staining, suggesting that modifications to reticulocytes as observed ex vivo may not happen as rapidly in vivo (Lim et al., 2016). In a separate study, it was also shown that *P. vivax* had normal growth and development in TfR1‐high reticulocytes in G6PD‐Mahidol mutants suggesting an advantage to invasion of reticulocytes in these settings (Bancone et al., 2017).

## 2 THE *Plasmodium vivax* RETICULOCYTE BINDING PROTEIN FAMILY

*P. vivax* invasion into reticulocytes is mediated by the *P. vivax* Reticulocyte Binding Protein (PvRBP) family. Genome sequencing of several *P. vivax* isolates identified 11 PvRBP family members that comprises of five full‐length genes (*pvrbp1a*, *pvrbp1b*, *pvrbp2a*, *pvrbp2b*, and *pvrbp2c*), three partial genes (*pvrbp1p1*, *pvrbp2p1*, and *pvrbp2p2*), and three pseudogenes (*pvrbp2d*, *pvrbp2e*, and *pvrbp3*) based on sequence homology to existing *P. vivax* RBP and *P. yoelli* Py235 members (Carlton et al., 2008; Gruner et al., 2004; Hester et al., 2013; Rayner et al., 2005; Rayner, Galinski, Ingravallo, & Barnwell, 2000). Full‐length genes encode large molecular weight proteins of over 280 kDa. Almost all PvRBPs have a signal peptide at the N‐terminus and a putative transmembrane domain at the C‐terminus. Transcriptome analyses show that several of the PvRBPs are expressed in field isolates (Bozdech et al., 2008).

The first functional study on PvRBPs showed that native PvRBP1a and PvRBP2c are expressed at the apical tip of *P. vivax* merozoites and form a high molecular weight complex that binds reticulocytes (Galinski, Medina, Ingravallo, & Barnwell, 1992). Recent studies using recombinant PvRBP proteins have further described their binding characteristics and defined regions of the proteins involved in binding red blood cells (summarised in Table 1). In particular, PvRBP1a has been particularly well characterized and is shown to bind preferentially to reticulocytes (Franca et al., 2016; Gupta et al., 2017; Han et al., 2016; Ntumngia et al., 2018). Although several studies have purified different PvRBP1a fragments, the collective results show that two of the best binding fragments range from residues 30–778 and 157–650 (Gupta et al., 2017; Ntumngia et al., 2018). In contrast, it has also been shown that PvRBP1a (residues 160–1170) binds normocytes (Franca et al., 2016). These conflicting results may indicate that in comparison with shorter recombinant fragments, PvRBP1a (residues 160–1170) may not be folded correctly and therefore not reflect its true binding properties. Recombinant PvRBP1b (residues 339–587), PvRBP2c (residues 464–876), and PvRBP2b (residues 168–1124) also showed preferential binding to reticulocytes (Franca et al., 2016; Gruszczyk et al., 2018; Gupta et al., 2017; Han et al., 2016), whereas recombinant PvRBP2a (residues 160–1135) has been shown to bind to both normocytes and reticulocytes (Franca et al., 2016).

**Table 1:** Binding Characteristics of PvRBPs

PvRBP	aa	Construct	Binding profile	Enzyme treatment	Reference
PvRBP1a	160–1170		Normocytes		Franca et al. (2016)
	Native protein		Reticulocytes		Galinski et al. (1992)
	Native protein		Reticulocytes	Nr, Ts, Cs	Gupta et al. (2017)
	30–778	rRBP1.1	Reticulocytes (34%)	Nr, Ts, Cs	Gupta et al. (2017)
	30–351	rRBP1.2	No binding		Gupta et al. (2017)
	352–778	rRBP1.3	Reticulocytes (~10%)		Gupta et al. (2017)
	352–599	rRBP1.4	Reticulocytes (~10%)		Gupta et al. (2017)
	1956–2315	rRBP1.5	No binding		Gupta et al. (2017)
	351–599		Reticulocytes		Han et al. (2016)
	352–599	rRBP1.4	Reticulocytes		Gupta et al. (2018)
	157–481	rRBP1:F7	No binding		Ntumngia et al. (2018)
	157–650	rRBP1:F8	Reticulocytes (~50%), Normocytes	Nr, Ts, Cs	Ntumngia et al. (2018)
	461–976	rRBP1:F4	Reticulocytes (~10–20%)		Ntumngia et al. (2018)
	632–976	rRBP1:F6	Reticulocytes (~10–20%)		Ntumngia et al. (2018)
	632–1078	rRBP1:F5	No binding		Ntumngia et al. (2018)
	950–1569	rRBP1:F1	Reticulocytes (~10–20%)		Ntumngia et al. (2018)
	1542–2192	rRBP1:F2	Reticulocytes (~10–20%)		Ntumngia et al. (2018)
	2162–2662	rRBP1:F3	Reticulocytes (~10–20%)		Ntumngia et al. (2018)
PvRBP1b	140–1275		Normocytes		Franca et al. (2016)
	339–587		Reticulocytes		Han et al. (2016)
PvRBP2a	160–1135		Normocytes and reticulocytes		Franca et al. (2016)
PvRBP2b	161–1454		Reticulocytes		Franca et al. (2016)
PvRBP2c	501–1300		No binding		Franca et al. (2016)
	Native protein		Reticulocytes		Galinski et al. (1992)
	Native protein		Reticulocytes	Nr, Tr, Cr	Gupta et al. (2017)
	168–524	rRBP2.1	Reticulocytes (10%)		Gupta et al. (2017)
	464–876	rRBP2.2	Reticulocytes (34%)	Nr, Tr, Cr	Gupta et al. (2017)
	2398–2736	rRBP2.3	No binding		Gupta et al. (2017)
PvRBP2‐P2	161–641		Normocytes and reticulocytes		Franca et al. (2016)

The use of recombinant PvRBP proteins have facilitated the determination of their functional properties; however, it would be important to examine if the respective native parasite proteins also display similar binding characteristics. Gupta and colleagues have also determined that the native PvRBP1a and PvRBP2c demonstrate the same binding profiles as has been previously shown by Galinski et al. (1992, Gupta et al., 2017). Experiments with native parasite proteins are extremely challenging for *P*. *vivax* research due to the absence of a long‐term in vitro culture system and limited access to clinical *P. vivax* samples. Nevertheless, it will be crucial to validate the binding profiles obtained with recombinant proteins with what is observed by using parasite lysates in which the PvRBP proteins are folded and functional for invasion.

## 3 NEW INVASION PATHWAY INTO RETICULOCYTES

Since the 1970s, it has been known that *P. vivax* invasion into red blood cells is dependent on Duffy Antigen Receptor for Chemokine (DARC; Miller, Mason, Clyde, & McGinniss, 1976). DARC is recognised by *P. vivax* Duffy Binding Protein (PvDBP) to mediate an essential step in *P. vivax* invasion, and many studies have contributed to the development of PvDBP as the lead *P. vivax* vaccine candidate, including the characterisation of anti‐PvDBP inhibitory antibodies that block invasion. However, DARC is present on both normocytes and reticulocytes and therefore cannot govern specific recognition of reticulocytes by *P. vivax*. Although it has been proposed that there is exposure of PvDBP binding site on DARC in young reticulocytes that allows it to selectively bind reticulocytes, this mechanism remains to be tested further by other laboratories (Ovchynnikova et al., 2017). Recent studies show that Transferrin Receptor 1 (TfR1, CD71) binds to PvRBP2b to mediate a critical pathway into reticulocytes (Gruszczyk et al., 2018; Gruszczyk, Kanjee, et al., 2018). TfR1 binds to its human ligand, iron‐loaded transferrin (Tf), and this complex regulates one of the main mechanisms for transporting iron into cells (Kawabata, 2019). TfR1 is highly expressed on reticulocytes and is selectively lost as they mature into normocytes. TfR1 mutant cells that are deficient in TfR1 expression have a strong defect in *P. vivax* invasion, showing that TfR1 mediates a critical invasion pathway into reticulocytes (Gruszczyk, Kanjee, et al., 2018). Mouse monoclonal antibodies against PvRBP2b also inhibit PvRBP2b binding to reticulocytes and block complex formation with TfR1. These anti‐PvRBP2b monoclonal antibodies also resulted in a reduction in parasite invasion using Thai and Brazilian clinical isolates (Gruszczyk, Kanjee, et al., 2018).

**Figure 1:**
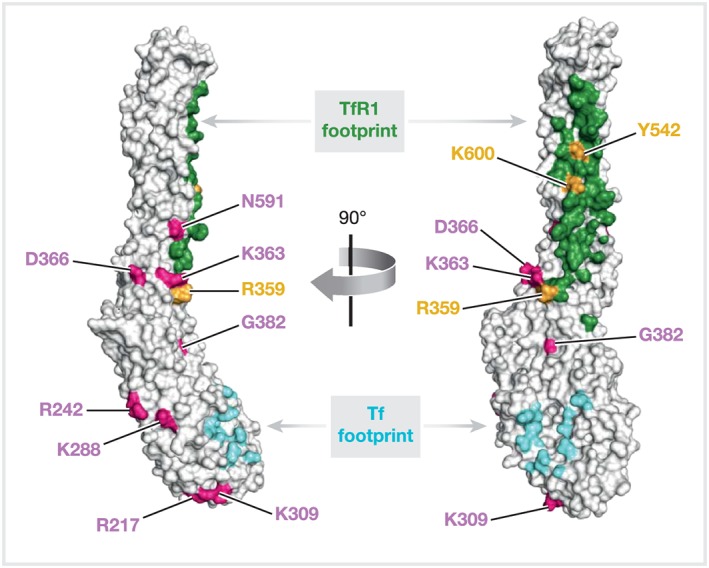
Surface representation of the cryo‐EM structure of PvRBP2b (168 to 633) shown in two orthogonal views. Regions interacting with TfR1 and Tf are shown in green and cyan, respectively. Field polymorphisms are labelled in pink and residues critical for TfR1 binding are labelled in orange

A 3.7 Å cryo‐electron microscopy (cryo‐EM) structure of the ternary complex of PvRBP2b, TfR1, and Tf has shed light on the critical residues involved in mediating complex formation (Gruszczyk, Huang, et al., 2018). The ternary complex is composed of homodimeric TfR1 (residues 120–760) bound to two molecules of iron‐loaded Tf (residues 1–679), with two molecules of PvRBP2b (residues 168–633) bound on either side. The most extensive interaction site is between PvRBP2b and TfR1, which has a surface buried area of ~1271 Å^2^, whereas the interaction site with Tf has a surface buried area of ~386 Å^2^. The region of PvRBP2b from 169 to 470 is highly polymorphic and under balancing selection (Gruszczyk, Huang, et al., 2018). Although this region binds to TfR1 and Tf, none of the prevalent field polymorphisms map to the amino acid residues on PvRBP2b that form critical contacts with either TfR1 or Tf as observed by the cryo‐EM structure (Figure 1). Extensive mutagenesis experiments identified three critical pairs of interacting residues for PvRBP2b‐TfR1 complex formation: those formed by PvRBP2b(Y542) and TfR1(Y211), a salt bridge formed between PvRBP2b(K600) and TfR1(E294), and a second salt bridge between TfR1(E149) and PvRBP2b(R359) (Figure 1).

At present, apart from TfR1, there has been no additional reticulocyte receptors that have been implicated as cognate receptors for PvRBPs. PvRBP1a binding is sialic acid independent but trypsin and chymotrypsin sensitive (Ntumngia et al., 2018), whereas PvRBP2c binding is insensitive to all three enzyme treatments (Gupta et al., 2017). These results strongly suggest that other reticulocyte receptors will be important for *P. vivax* invasion

## 4 TfR1 AS A CELLULAR RECEPTOR FOR NEW WORLD HAEMORRHAGIC FEVER ARENAVIRUSES

TfR1 is also a cellular receptor for human New World haemorrhagic fever arenaviruses, including Machupo (MACV), Junin, Guanarito, and Sabiá viruses (Abraham, Corbett, Farzan, Choe, & Harrison, 2010; Radoshitzky et al., 2007). Residues 208 to 212 of the TfR1 apical domain provide a critical recognition site for these viruses (Abraham et al., 2010). Of particular interest, Y211 that is localized in the apical domain of TfR1 is a critical residue for entry of these New World haemorrhagic arenaviruses and *P. vivax* (Abraham et al., 2010; Gruszczyk *et al*., 2018). Furthermore, the binding of recombinant PvRBP2b can compete with the binding of recombinant MACV Glycoprotein 1 to TfR1, showing that these two pathogens have co‐opted a similar site on the apical domain of TfR1 for entry into their target cells (Gruszczyk, Kanjee, et al., 2018).

## 5 STRUCTURAL SCAFFOLDS OF PvRBPs

The first description of a family of Reticulocyte or Normocyte binding proteins (RBP or NBP) was described in *P. yoelli* as the Py235 proteins (reviewed in Gruner et al., 2004). The description of the Py235 and the PvRBP family led to the identification of the homologous *P. falciparum* Reticulocyte‐binding Homolog (PfRh) family by gene structure and sequence similarity (Rayner et al., 2000; Triglia et al., 2001). The PfRh family consists of several members PfRh1, PfRh2a, PfRh2b, PfRh4, PfRh5, and the pseudogene PfRh3, and many of these members have been shown to be important in red blood cell invasion (Chen et al., 2011; Crosnier et al., 2011; Lopaticki et al., 2011; Tham et al., 2010; Weiss et al., 2015). Two red blood cell receptors have been identified to bind to PfRh proteins; PfRh5 binds to Basigin (otherwise known as CD147, EMMPRIN) and PfRh4 binds to Complement Receptor 1 (CR1) (Crosnier et al., 2011; Tham et al., 2010).

PfRh5 cannot be deleted in any strain (Baum et al., 2009; Hayton et al., 2008), suggesting that it would be an ideal component targeting the blood stages in an effective *P. falciparum* vaccine. PfRh5 is smaller than the other PfRh members and does not contain a transmembrane region (Baum et al., 2009; Hayton et al., 2008). During parasite invasion, PfRh5 forms a complex with *P. falciparum* PfRh5‐interacting protein (PfRipr; Chen et al., 2011) and the cysteine‐rich protective antigen (PfCyRPA; Dreyer et al., 2012; Reddy et al., 2015). The PfRipr/CyRPA/PfRh5‐Basigin complex is required for triggering the release of Ca^2+^ and establishing the tight junction (Volz et al., 2016). These observations show that the PfRh5/PfRipr/CyRPA complex is essential in the sequential molecular events leading to *P. falciparum* invasion of human red blood cells.

In *P. vivax* and *P. knowlesi*, the exact homologue to PfRh5 remains elusive, but the homologs for PvRipr and PvCyRPA have been identified (Hoo et al., 2016). In a recent study, *P. knowlesi* PkRipr and PkCyRPA were shown to be essential for parasite viability (Knuepfer et al., 2019). Furthermore, PkRipr did not form a complex with PkCyRPA, but instead formed a trimeric complex with thrombospondin‐related anonymous protein (PkPTRAMP) and cysteine‐rich small secreted protein (PkCSS). Conditional knockout of any component of the trimeric complex resulted in merozoites that could attach to human erythrocytes, but were unable to invade. These results suggest that Ripr and CyRPA have different roles in *P. knowlesi* than in *P. falciparum* and may also have different roles in *P. vivax* invasion.

**Figure 2:**
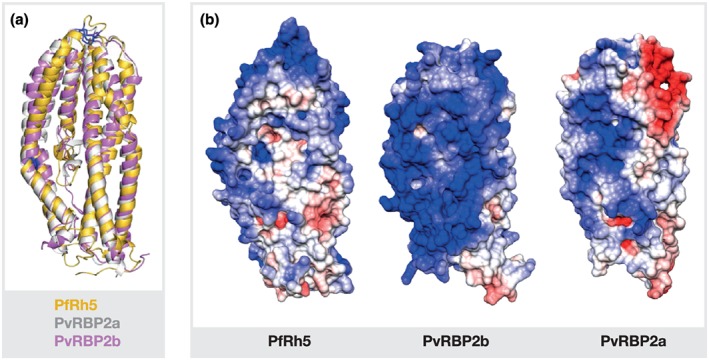
Structural comparison of PvRBP2a, PvRBP2b and PfRh5. (a) Superposition of PvRBP2a (PDB ID 4Z8N, grey), PvRBP2b (PDB ID 5W53, pink), and PfRh5 (PDB ID 4WAT, yellow) structures shown in ribbon representation. The position of two disulfide bridges (indicated by blue) is conserved in all three structures. (b) Surface charge distribution of PvRBP2a, PvRBP2b, and PfRh5. The electrostatic surface potentials were calculated using the programs PDB2PQR and APBS in Chimera with the non‐linear Poisson–Boltzmann equation and contoured at ±5 kT/e. Negatively charged surface areas are coloured in red, positively charged surface areas in blue

The crystal structure of the N‐terminal domain of PvRBP2b closely resembles the structures of the homologous domains of PvRBP2a and PfRh5 (Chen et al., 2014; Wright et al., 2014a; Gruszczyk et al., 2016; Gruszczyk, Kanjee, et al., 2018). Structural comparisons of PfRh5, PvRBP2a and PvRBP2b show that the placements of two disulfide bridges are conserved (Figure 2; Chen et al., 2014, Gruszczyk et al., 2016, Wright, Hjerrild, Bartlett, Douglas, et al., 2014a). The first bonded pair of cysteine residues between C345 and C351 in PfRh5 overlaps with C299 and C303 in PvRBP2a and with C312 and C316 in PvRBP2b and links a loop at the tip of the domain. The second bonded pair cysteine residues between C224 and C317 in PfRh5 overlap with C227 and C271 in PvRBP2a and with C240 and C284 in PvRBP2b and connects two antiparallel α‐helices (α2b, α3a) in the middle of the domain in each case. Although these domains have similar α‐helical scaffolds, they bind different receptors: PvRBP2b to TfR1 and PfRh5 to Basigin (Crosnier et al., 2011; Gruszczyk, Kanjee, et al., 2018; Wright, Hjerrild, Bartlett, Douglas, et al., 2014a). In contrast to PfRh5 that receptor recognition site is located on the tip of the domain, binding of PvRBP2b to TfR1‐Tf involves residues located at the side of the domain and residues of the adjacent α‐helical domain of this protein. The receptor for PvRBP2a is currently unknown.

The surface charge distribution of PvRBP2a, PvRBP2b, and PfRh5 is different (Figure 2). In particular, PvRBP2a has a distinctive negative patch on the apex of the molecule that corresponds to the Basigin‐binding area of PfRh5, whereas PfRh5 as well as the N‐terminal domain of PvRBP2b mostly displays a positive charged surface (Gruszczyk, Kanjee, et al., 2018; Wright, Hjerrild, Bartlett, Douglas, et al., 2014a). The differences in surface charge may be important in mediating the specific interactions between these parasite adhesins and their respective red blood cell receptors.

## 6 ANTIBODIES TO PvRBP2b AND PfRh5

**Figure 3:**
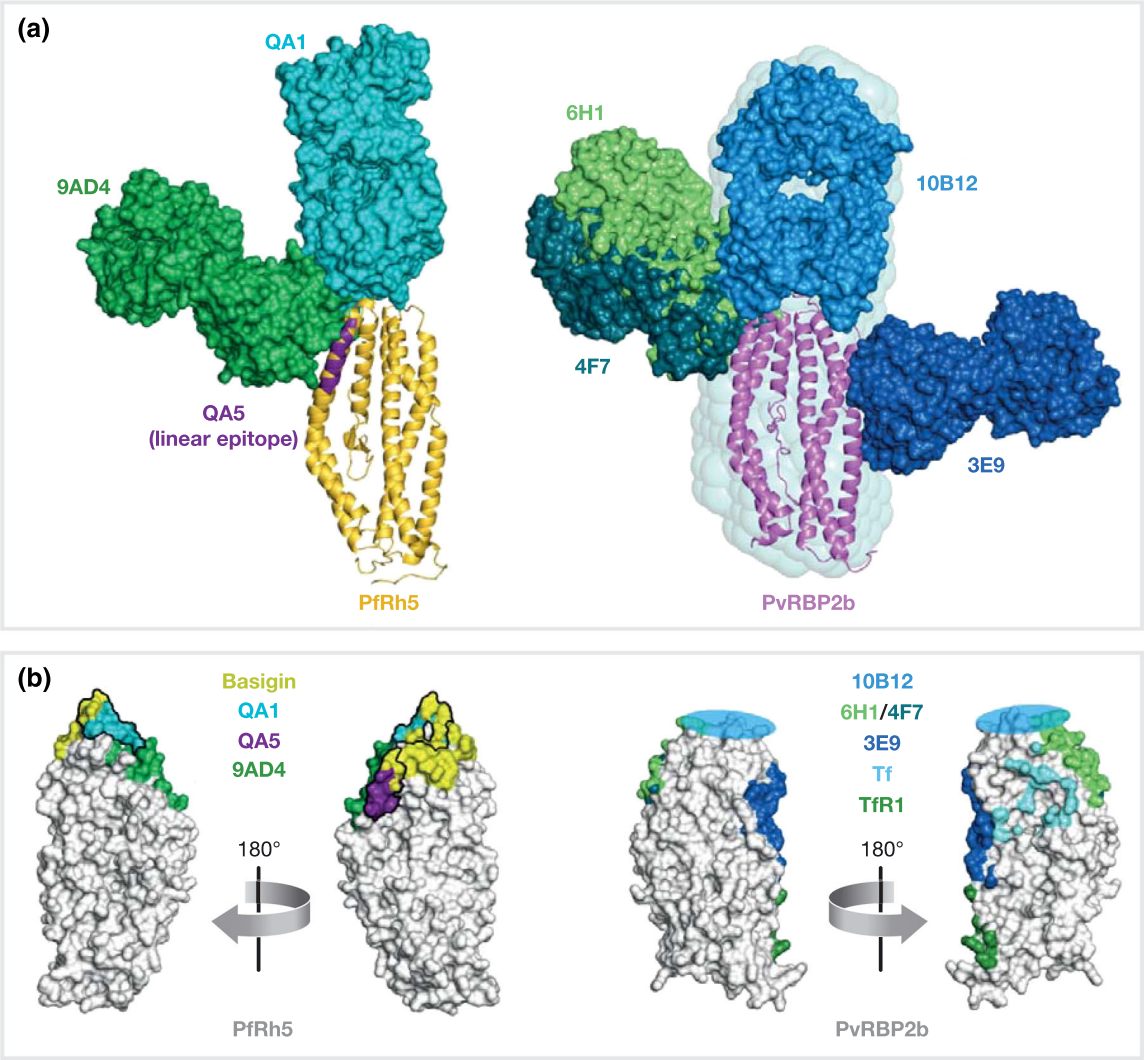
Epitopes of inhibitory mouse antibodies against PfRh5 and PvRBP2b. Structures of antigen‐Fab complexes are superimposed on the corresponding antigen. (a) Antigens PfRh5 (PDB ID 4WAT, yellow) and PvRBP2b (PDB ID 5W53, pink) are shown in ribbon representation, Fabs (each with heavy and light chains in the same colour) are in surface representation. The linear epitope of inhibitory antibody QA5 (PfRh5 residues 201–213) is indicated in purple. The SAXS‐derived model of the PvRBP2b‐10B12 complex is surrounded by its SAXS‐envelope. (b) Surface representation of PfRh5 and PvRBP2b (white). Antibody and receptor footprints are coloured with a 6 Å‐distance cut‐off. The rough binding region of 10B12 is indicated by a blue circle. Antibody epitopes of QA1 and QA5 directly overlap with the Basigin binding site of PfRh5

Antibodies against PvRBPs and antibodies against PfRh family members are known to inhibit parasite invasion. Several antibody binding regions critical for receptor–ligand interactions have been identified on PfRh5 and PvRBP2b by X‐ray crystallography and small‐angle X‐ray scattering (SAXS; Figure 3; Wright, Hjerrild, Bartlett, Douglas, et al., 2014a; Gruszczyk *et al*., 2018). Crystal structures of PfRh5 complexed with the antigen binding fragments (Fab) of mouse antibodies QA1 and 9AD4, respectively, revealed distinct conformational epitopes (Wright, Hjerrild, Bartlett, Douglas, et al., 2014a). A linear epitope of another inhibitory antibody, QA5, was identified by peptide mapping, and the binding region was confirmed by SAXS. Binding sites of QA1 and QA5 that are able to inhibit Rh5‐Basigin binding in vitro overlap with different parts of the receptor binding site on PfRh5. The 9AD4 antibody does not interfere with in vitro PfRh5‐binding to Basigin, and its epitope is next to the receptor binding site but does not overlap. The ability of this antibody to inhibit parasite growth is probably caused by a steric hindrance of the interaction of membrane‐tethered PfRh5 and Basigin.

For PvRBP2b, four mouse monoclonal antibodies (3E9, 4F7, 6H1, and 10B12) are able to inhibit PvRBP2b binding to reticulocytes. Three of these 3E9, 6H1, and 10B12 were able to block PvRBP2b‐TfR1 complex formation and *P. vivax* invasion in Thai and Brazilian clinical isolates (Gruszczyk *et al*., 2018). Co‐crystal structures of 3E9, 4F7, and 6H1 with PvRBP2b, as well as a SAXS derived model of 10B12 binding to PvRBP2b, revealed that these antibodies recognise different structural epitopes, all located in the N‐terminal domain of PvRBP2b (Gruszczyk *et al*., 2018). The antibodies 4F7 and 6H1 have overlapping epitopes, both bind to the side of PvRBP2b. Their binding sites on PvRBP2b map to a similar area as the binding site of 9AD4 on PfRh5 (Figure 3). All four anti‐PvRBP2b antibodies do not completely overlap with the receptor recognition site but would either sterically clash with TfR1‐Tf binding of PvRBP2b (3E9) or inhibit receptor engagement through steric hindrance with the reticulocyte membrane (4F7, 6H1, and 10B12).

Similar to anti‐PfRh5 antibody QA1, antibody 10B12 engages its antigen at the tip of the N terminal domain, probably also facing the loop which is stabilised by a disulfide bond (Gruszczyk *et al*., 2018). The presence of the corresponding disulfide bridge and loop of PvRBP2a is also required for the protein function as mutation of C299 and C303 to serine residues results in a loss of the capability of PvRBP2a to bind erythrocytes while not altering the overall structural conformation of the protein (Gruszczyk et al., 2016). Collectively, these results highlight the importance of the disulfide linked loop at the apex of PvRBP and PfRh red blood cell binding domains as a target for inhibitory antibodies. Future investigations should explore conformational epitopes as immunogens that may generate cross‐inhibitory antibodies that inhibit PvRBP and PfRh function in parasite invasion.

In addition to the anti‐PvRBP2b antibodies, several studies have examined the role of antibodies to other PvRBPs in blocking parasite invasion or red blood cell binding. Natural human antibodies against PvRBP1a and PvRBP2c have been shown to inhibit reticulocyte binding (Gupta et al., 2017), but the evidence for inhibition of *P. vivax* invasion is lacking. Rabbit antibodies targeting PvRBP1a (residues 352–599) were recently shown to have no significant impact on *P. vivax* invasion (Gupta et al., 2018); however, the failure to reach statistical significance may be due to the large variation in the inhibition efficacy across the few biological replicates. The large variation of results obtained from *P. vivax* invasion assays from different clinical isolates remains the one of the main challenges in understanding the importance of any particular *P. vivax* invasion ligand.

## 7 NEW MODEL SYSTEMS

New model systems using other malaria species may facilitate anti‐PvRBP antibody screening and accelerate the pace of vaccine development for these proteins (Martinelli & Culleton, 2018). Recently, transgenic *P. knowlesi* parasites genetically modified to express PvDBP were used successfully to assess the efficacy of anti‐PvDBP human monoclonal antibodies (Mohring et al., 2019; Rawlinson et al., 2019). However, the respective RBP ligands in *P. knowlesi*, which are *P. knowlesi* Normocyte Binding Proteins (NBPXa and NBPXb), are strongly divergent from PvRBPs. PkNBPXa is required for the *P. knowlesi* growth and replication in human red blood cells, as deletion of this gene restricts the growth of *P. knowlesi* to monkey red blood cells (Moon et al., 2013; Moon et al., 2016). It should be noted that PkNBPXa and PkNBPXb are both found as pseudogenes in the reference genomes of *P. vivax* as PvRBP3 and PvRBP2e. However, sequencing of a *P. vivax* field isolate has identified an intact copy of PvRBP2e (Hester et al., 2013). The distinct RBPs or NBP that are expressed in *P. vivax* or *P. knowlesi* may actually govern why one is restricted to reticulocytes and the other not. It would be interesting if the transgenic lines expressing different PvRBPs in a *PkNBPXa*‐null background could “complement” the deletion and thus allow entry of *P. knowlesi* into human red blood cells. If any expressed transgenic PvRBP is able to rescue the PkNBPxa‐null phenotype, this would imply that the respective PvRBP is expressed and functional in *P. knowlesi* and provide an important model to determine the functional activity of anti‐PvRBP antibodies. *P. cynomolgi* is the sister taxon to *P. vivax* and possesses multiple PvRBP homologues with most showing a high degree of sequence identity (PcRBPs; Tachibana et al., 2012). Substantial progress has been made to establish a robust *P. cynomolgi* culture in monkey cells that would allow a short‐term invasion assays into human reticulocytes (Kosaisavee et al., 2017; Zeeman, der Wel, & Kocken, 2013). Although *P. cynomolgi* is able to enter monkey red cells of all ages, it has a strict preference for TfR1 and DARC‐positive reticulocytes in the presence of human red blood cells (Kosaisavee et al., 2017). To determine if *P. cynomolgi* would be a useful system to examine the function of anti‐PvRBP antibodies, it would be important to determine if the PcRBP homologs have the same molecular function as their respective PvRBPs and whether anti‐PvRBP antibodies recognise different members of PcRBPs. Both transgenic *P. knowlesi* and *P. cynomolgi* cultures are promising models to complement the characterization of inhibitory antibodies to *P. vivax* ligands and facilitate the understanding of the mechanisms of *P. vivax* invasion.

## 8 NATURALLY ACQUIRED ANTIBODY RESPONSE TO PvRBPs

The study of the human immune response to malaria infection is central to the discovery of new vaccine candidates and biological markers of disease. The availability of the *P. vivax* parasite genome since 2008 has enabled researchers to examine a much larger panel of proteins (Baum et al., 2016; Finney et al., 2014; Franca et al., 2017; Hostetler et al., 2015; Longley et al., 2017), not least the entire family of PvRBPs. Although these studies show that many other parasite antigens that are associated with protection, we will only focus on the results for PvRBPs for this review. To date, all PvRBPs have been subjected to serological examinations and are all shown to be targets of natural antibody responses (Franca et al., 2016; Longley, White, et al., 2017).

Plasma samples from different endemic regions have been used to study natural acquisition of antibodies to PvRBPs, from the relatively high transmission areas in India (Gupta et al., 2017) and Papua New Guinea (PNG; Franca et al., 2016; Ntumngia et al., 2018) to the lower transmission sites in Brazil (Ferreira et al., 2014; Longley et al., 2018; Ntumngia et al., 2018; Tran et al., 2005), Cambodia (Hostetler et al., 2015), Colombia (Rojas Caraballo, Delgado, Rodriguez, & Patarroyo, 2007), Korea (Han et al., 2015; Han et al., 2016), Solomon Islands (Longley et al., 2018), and Thailand (Hietanen et al., 2015; Longley et al., 2017; Longley et al., 2018). These samples encompassed specimens from uninfected endemic residents, infected asymptomatic *P. vivax* carriers, and acute *P. vivax* patients. The diversity of these samples provides rich data for identifying new vaccine candidates as well as serological markers of *P. vivax* malaria.

PvRBP1a was the first protein in the PvRBP family whose antibody response was systematically examined (Tran et al., 2005). In this initial study published in 2005, plasma specimens from the Brazilian Amazon state of Rondonia were tested against five recombinant PvRBP1a fragments, which together span nearly the entire extracellular domain (>2,600 amino acids). The study revealed key features of the natural antibody response that appear to be common among PvRBPs, including (a) positive correlation between the antibody level and parasite exposure, (b) IgG subtypes being biased towards cytophilic IgG1 and IgG3, and (c) association between the antibody level and clinical protection. To date, PvRBP1a is the only full‐length PvRBP whose entire extracellular sequence has been mapped serologically. Comparison of the IgG responses across different subdomains of PvRBP1a revealed stronger reactivity towards the N‐terminal half, a finding which was later confirmed in a separate study (Ntumngia et al., 2018). This N‐terminal bias coincides with a higher degree of genetic diversity of the N‐terminal domain (Rayner et al., 2005), suggesting a stronger immunogenic selection pressure on this region of the protein.

In the endemic populations, the magnitudes of IgG responses to different PvRBPs are generally correlated (Gupta et al., 2017; Hietanen et al., 2015) and tend to increase with age (Hietanen et al., 2015; Longley et al., 2017). This co‐acquisition of antibodies to different PvRBPs is unlikely due to cross‐reactivity given the limited sequence similarity between these proteins. Not surprisingly, human IgG obtained by affinity purification with PvRBP1a showed little cross‐reactivity against PvRBP2c, and vice‐versa (Gupta et al., 2017). Rabbit immunizations with recombinant PvRBPs also generated antibodies specific to the respective antigen (Franca et al., 2016). Therefore, the co‐development of IgG responses to different PvRBPs is likely due to repeated *P. vivax* infections that expose the human body to all proteins simultaneously. In addition to age, other measures of parasite exposure such as the number of previous malaria episodes (Tran et al., 2005), years of residence in endemic areas (Tran et al., 2005), and the estimated number of new infections acquired in the lifetime (Franca et al., 2016) have been shown to positively associate with anti‐PvRBP reactivities.

## 9 SEROLOGICAL MARKERS OF EXPOSURE

People with concurrent *P. vivax* infection, both symptomatic and asymptomatic, tend to have higher levels and breadth of IgG responses to PvRBPs than do uninfected individuals (Franca et al., 2017; Longley, França, et al., 2017). The heightened sero‐reactivities in infected individuals suggest that PvRBPs may be viable markers for comparing malaria burdens in endemic populations. Indeed, the IgG responses to PvRBP1a and PvRBP2c fragments have been shown to map spatial heterogeneity in western Thailand (Longley et al., 2017) and be able to differentiate different endemic communities in Brazilian Amazon (Tran et al., 2005). The magnitudes of IgG responses to several PvRBPs were also found to decline after clearance of infection, with estimated half‐lives of 2–18 months (Longley et al., 2017). Therefore, antibodies to some of these proteins, especially the short‐lived ones, may be good surrogates of recent exposure. In a recent attempt to develop a panel of serological markers of recent infections (Longley et al., 2018), a recombinant fragment of PvRBP2b was the best performing antigen among the >300 *P. vivax* proteins tested. The IgG response to this protein has the sensitivity and selectivity of 74% in detecting *P. vivax* infection within the previous 9 months. Combining PvRBP2b with other proteins further improved the diagnostic performance, making antibody‐based surveillance an attractive approach for malaria control and elimination programs (Greenhouse et al., 2019).

## 10 SEROLOGICAL MARKERS OF PROTECTION

Identification of parasite proteins associated with clinical protection is an approach often taken in malaria vaccine discovery (Cutts et al., 2014; Franca et al., 2017). Because naturally acquired immunity against malaria is gained through repeated infections, an association between the antibody level and protection is often confounded by exposure, a factor that has to be accounted for in sero‐epidemiological analysis. The most comprehensive study to examine association between the IgG reactivities to PvRBPs and clinical protection (Franca et al., 2016) came from a cohort of young children in East Sepik, PNG, whose exposure to *P. vivax* was defined by molecular force of blood stage infection (_mol_FOB, the incidence of new blood infection). In this study, children 1–3 years old were followed for up to 16 months, with blood sampling every 8 weeks and at clinical malaria episodes. *P. vivax* infections were detected by PCR, and the parasites were genotyped for determination of _mol_FOB. After adjusting for exposure, IgG for each of the six PvRBPs tested (PvRBP1a, PvRBP1b, PvRBP2a, PvRBP2b, PvRBP2c, and PvRBP2p2) was associated with protection, with a reduction of 30–50% in risk of *P. vivax* malaria observed in children who had medium to high antibody levels. However, in multivariate analysis to account for co‐acquisition of antibodies to different PvRBPs, only IgG to PvRBP1a and PvRBP2b remained, with IgG1 being the underlying antibody subclass associated with protection. Notably, these two proteins were amongst the strongest protective antigens in the panel of 38 *P. vivax* antigens tested with the same methodology (Franca et al., 2017). Nevertheless, it is important to note that these studies were only performed with recombinant proteins, which may have inherent caveats such as the use of a single variant and that the protein folding may be different from native protein. In particular, for proteins that are highly polymorphic, immune responses to a different variant may be very different. Consistently, the magnitude of IgG to PvRBP1a fragments had previously been associated with duration since the last known malaria episode, a proxy of protection, in a Brazilian cohort (Tran et al., 2005). The magnitude of IgG response to PvRBP2b had also been associated with reduced parasitaemia in Thai adult patients independently of age (Hietanen et al., 2015). Thus, among the PvRBPs, PvRBP1a and PvRBP2b appear to be the strongest vaccine candidates.

## 11 CONCLUSIONS

The PvRBP family of *P. vivax* parasite adhesins is clearly emerging to be important for reticulocyte invasion and is the target of naturally acquired immunity in many different transmission settings. Future studies on the identification of other reticulocyte receptors for PvRBP and the characterization of inhibitory antibodies will provide insightful information on the multiple invasion pathways of *P. vivax* and how to target them to block entry into reticulocytes.
